# Unraveling the immune evasion mechanisms in the tumor microenvironment of head and neck squamous cell carcinoma

**DOI:** 10.3389/fimmu.2025.1597202

**Published:** 2025-05-14

**Authors:** Xuri Zhao, Yaping Zhu, Youya He, Weiyan Gu, Qi Zhou, Bei Jin, Shenguo Chen, Haisheng Lin

**Affiliations:** Department of Stomatology, Taizhou Hospital of Zhejiang Province Affiliated to Wenzhou Medical University, Taizhou, Zhejiang, China

**Keywords:** head and neck squamous cell carcinoma, tumor microenvironment, immune escape, immunotherapy, immune checkpoint, metabolic reprogramming

## Abstract

Head and neck squamous cell carcinoma (HNSCC) is a highly aggressive malignancy characterized by a complex tumor microenvironment (TME) that plays a pivotal role in tumor initiation, progression, and immune evasion. Recent advancements have highlighted the intricate interplay between immune cell infiltration patterns, immune checkpoint dysregulation, and metabolic reprogramming in driving HNSCC immune escape. Despite these insights, significant challenges remain, including the incomplete understanding of specific immune evasion pathways and the lack of personalized therapeutic strategies. To address these gaps, this review introduces a novel “Trinity” regulatory network of immune evasion in HNSCC, encompassing: (1) metabolic reprogramming-mediated immune checkpoint modulation, (2) stromal cell-driven immune dysfunction, and (3) epigenetic remodeling fostering immune tolerance. This framework provides a theoretical foundation for the development of multi-targeted combination therapies and offers innovative strategies to overcome immune evasion. Additionally, this review systematically synthesizes the current understanding of the relationship between the HNSCC microenvironment and immune escape, with a focus on emerging immunotherapeutic approaches such as PD-1/PD-L1 inhibitors and CAR-T cell therapy. Leveraging cutting-edge single-cell sequencing and spatial transcriptomics, we elucidate the spatiotemporal heterogeneity of the HNSCC immune landscape and propose a new paradigm of “lineage plasticity-driven immune adaptation.” These insights not only advance our understanding of HNSCC biology but also pave the way for the development of precision immunotherapies aimed at improving patient survival and quality of life. By integrating multidisciplinary perspectives, this work underscores the importance of targeting the TME to achieve durable clinical responses and overcome immunotherapy resistance in HNSCC.

## 1 Introduction

Head and neck squamous cell carcinoma (HNSCC) is a prevalent malignant tumor with rising incidence and mortality rates globally (1). The tumor microenvironment (TME)—comprising immune cells, fibroblasts, vasculature, microbiota, and extracellular matrix—plays a decisive role in tumor initiation, progression, and resistance to therapy. Immune evasion within this complex ecosystem has emerged as a key hallmark of HNSCC (2, 3). A central driver of these processes is immune evasion, a critical strategy by which tumors circumvent host immune surveillance. In HNSCC, diverse TME-associated factors—including cytokines, immunosuppressive cells, and intricate intercellular communication networks—are closely linked to immune evasion.

Within the HNSCC TME, immune cell infiltration patterns profoundly influence tumor progression and immune evasion. Studies demonstrate that CD8+ T cells and regulatory T cells (Tregs) play dual roles in immune surveillance and suppression. High CD8+ T cell infiltration correlates with favorable prognosis, whereas elevated Treg levels are associated with immunosuppression, potentially enabling tumor cells to evade immune attack (4). Tumor-associated macrophages (TAMs) further contribute to immune escape in pan-cancer (5) not to mention HNSCC; their M2-polarized phenotype promotes tumor growth, metastasis, and suppression of antitumor immunity (6).

Metabolic reprogramming in HNSCC represents another crucial axis of immune evasion. Tumor cells alter metabolic pathways to exploit nutrients for proliferation while impairing immune cell function. For instance, excessive lactate production by tumor cells induces localized acidosis, thereby inhibiting T cell activation and proliferation (7). Hypoxic conditions within the TME further exacerbate immune escape by undermining immune surveillance (8).

Immune checkpoint dysregulation is a hallmark of HNSCC immune evasion. Tumor cells upregulate PD-L1 and other checkpoint molecules to suppress T cell activity and evade immune detection (9), and it is even closely related to cell death(Li, 10). Although immune checkpoint inhibitors (e.g., PD-1/PD-L1 blockers) have emerged as a cornerstone of HNSCC therapy, their clinical efficacy remains limited to a subset of patients, reflecting the complexity and heterogeneity of the TME in modulating treatment responses (11).

Despite progress in dissecting individual mechanisms—such as immune checkpoint overexpression, T cell exhaustion, and stromal remodeling—the field lacks an integrated framework to explain how these factors collectively drive immune escape. To address this, we propose a “Trinity” model involving three interlinked pathways: (1) metabolic reprogramming, (2) stromal cell-driven immune dysfunction, and (3) epigenetic remodeling.

This review is the first to systematically integrate and elucidate the spatiotemporal heterogeneity of the HNSCC immune microenvironment, proposing a novel paradigm of “lineage plasticity-driven immune adaptation.” And we synthesize current knowledge on HNSCC immune escape mechanisms and provide new theoretical and practical insights based on the “Trinity” network. By innovatively synthesizing the network regulation of immune metabolism, checkpoint interactions, and cellular crosstalk, this work provides fresh insights into the multifaceted mechanisms of immune evasion in HNSCC. Furthermore, it establishes a theoretical foundation for developing precision combination therapies targeting TME-specific vulnerabilities.

## 2 Immune infiltration patterns in the HNSCC microenvironment

### 2.1 Cellular heterogeneity of immune infiltration landscapes

**Figure 1:**
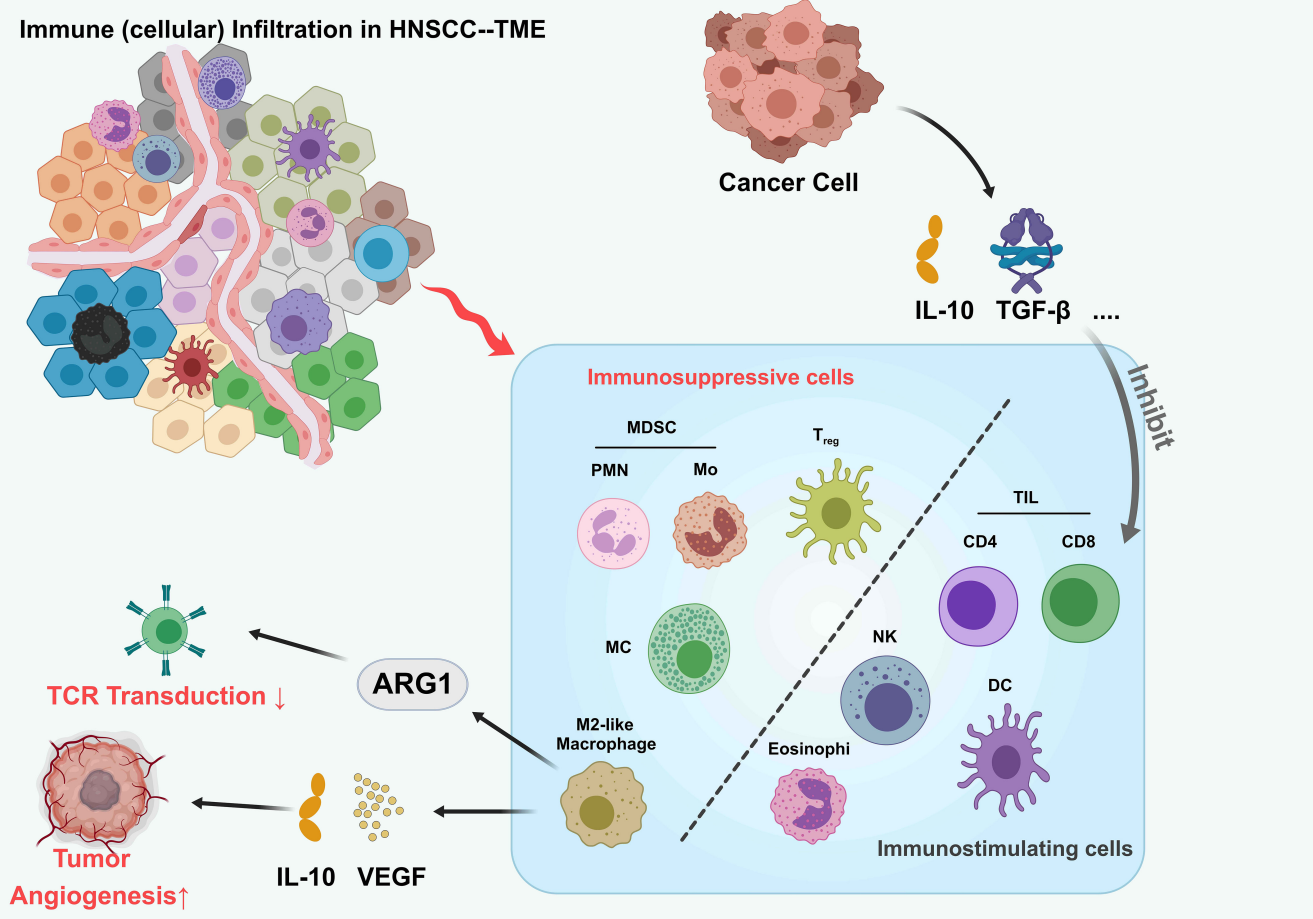
Tumor immune infiltrating microenvironment of HNSCC. The tumor microenvironment (TME) in head and neck squamous cell carcinoma (HNSCC), highlighting interactions between immune cells and cancer cells. Tumor cells secrete immunosuppressive cytokines (IL-10, TGF-β) that recruit myeloid-derived suppressor cells (MDSCs; PMN and Mo subsets), regulatory T cells (Treg), M2-like macrophages, and eosinophils. These cells inhibit tumor-infiltrating lymphocytes (TILs), including CD4+, CD8+ T cells, natural killer (NK) cells, and dendritic cells (DCs), suppressing antitumor immunity. ARG1 expression in MDSCs and M2-like macrophages further dampens T cell receptor (TCR) signaling. Additionally, VEGF secretion by tumor cells promotes angiogenesis. This balance between immunosuppressive and immunostimulatory cells shapes the TME, influencing tumor progression.

The HNSCC microenvironment exhibits highly heterogeneous and complex immune cell infiltration patterns (12) (**Figure 1**). Key infiltrating immune populations include CD8+ T cells, CD4+ T cell subsets (Th1/Th2/Th17), natural killer (NK) cells, macrophages, and dendritic cells, collectively forming a dynamic immune network that governs tumor progression and therapeutic responses (13). CD8+ T cells, as central antitumor effectors, demonstrate prognostic significance linked to their infiltration density and spatial organization (14). However, tumor-derived immunosuppressive factors such as IL-10 and TGF-β drive CD8+ T cell dysfunction and exhaustion, facilitating immune escape (15). TAMs further contribute to this immunosuppressive milieu. While M1-polarized TAMs exhibit antitumor activity, M2-polarized TAMs dominate the HNSCC microenvironment, promoting tumor progression via ARG1-mediated L-arginine depletion (impairing TCR signaling) and IL-10/VEGF-driven immunosuppressive angiogenesis (16, 17).

### 2.2 Tumor-immune cell crosstalk

Bidirectional interactions between tumor and immune cells critically shape HNSCC progression and therapeutic resistance. Programmed death-ligand 1 (PD-L1), overexpressed in ~50% of HNSCC cases, mediates immune evasion through dual mechanisms: membrane-bound PD-L1 engages PD-1 on T cells to induce exhaustion, while extracellular vesicle (EV)-encapsulated PD-L1 systemically suppresses T cell activity (18). Spatial proteomic analyses reveal PD-L1 enrichment at invasive fronts, particularly on cancer stem-like cells (CSCs), where PD-1/PD-L1 interactions impair immune synapse formation (19). Spatial proteomic analyses reveal PD-L1 enrichment at invasive fronts, particularly on CSCs, where PD-1/PD-L1 interactions impair immune synapse formation (20). Tumor-derived IL-6 and TGF-β synergistically reinforce immunosuppression: IL-6 activates STAT3 to upregulate B7-H3 expression, while TGF-β drives Treg differentiation and confers CD8+ T cells with stem-like exhausted epigenetic states (21). Myeloid-derived suppressor cells (MDSCs) exacerbate immune evasion through peroxynitrite-mediated antigen modification, impairing TCR recognition (22).

### 2.3 Multilayered regulation of microenvironmental niches

Immune infiltration patterns are modulated by tumor-intrinsic mutations, cytokine networks, and physicochemical stressors. High tumor mutational burden (TMB) inversely correlates with immune infiltration, suggesting genomic instability shapes immune evasion (23). NOTCH1 mutations disrupt CD8+ T cell/Treg balance, while PIK3CA-activating mutations recruit MDSCs via CXCL12/CXCR4 signaling (24). In addition, the hypoxic state in the tumor microenvironment also suppresses the function of immune cells, leading to immune escape (25). The study also found that tumor-associated fibroblasts (CAFs) play an important role in the HNSCC microenvironment (26–28). CAFs alter the infiltration pattern of immune cells and promote tumor growth and metastasis by secreting a variety of cytokines and growth factors (29). Hypoxia-driven HIF-1α activation reprograms Treg metabolism to enhance adenosine production via CD39/CD73 upregulation, while extracellular acidosis (pH 6.5–6.8) inhibits NFAT nuclear translocation, blunting T cell activation (30). CAFs emerge as central stromal orchestrators, secreting TGF-β superfamily ligands to polarize M2 macrophages and LOXL2-mediated collagen crosslinking to form T cell-excluding physical barriers (31).

### 2.4 Spatial heterogeneity and immune desert/excluded phenotypes

In recent years, the breakthrough of single-cell spatial analysis technology has enabled the detailed analysis of the spatial structure of HNSCC immune microenvironment. Spatial transcriptomic studies have shown that HNSCC has a characteristic “Immune Desert” and “Immune Excluded” phenotype (32). The immune desert region showed substantial loss of effector T cells and dendritic cells, forming a “cold tumor” characteristic without immune monitoring. CD8+ T cells and TAMs were abundant in the immune rejection region, but these cells showed a state of functional inactivation, and their spatial distribution was highly coexisting with the remodeled ECM region (33). This spatial heterogeneity suggests that tumor cells establish hierarchical immune escape mechanisms through physical barrier construction and immunosuppressive signal diffusion.

Pseudotemporal analysis reveals spatial dynamics of T cell exhaustion, with CD8+ T cells transitioning from TCF1+ progenitor states at tumor margins to TIM-3+ terminally exhausted populations in cores—a process governed by TOX/OX40-driven epigenetic reprogramming (34, 35).

Current research paradigms are shifting from targeting oncogenic drivers toward remodeling the immunosuppressive niche. Deciphering the stromal barriers in immune deserts and metabolic suppression networks in excluded zones will enable spatiotemporally precise combination strategies (e.g., ECM degradation coupled with checkpoint blockade). This therapeutic evolution—from tumor eradication to immune ecosystem reconstruction—holds promise for overcoming the spatial limitations of current immunotherapies and achieving durable clinical responses.

## 3 Dynamic expression and regulatory networks of immune checkpoints

### 3.1 Spatiotemporal regulation of the PD-1/PD-L1 axis

**Figure 2:**
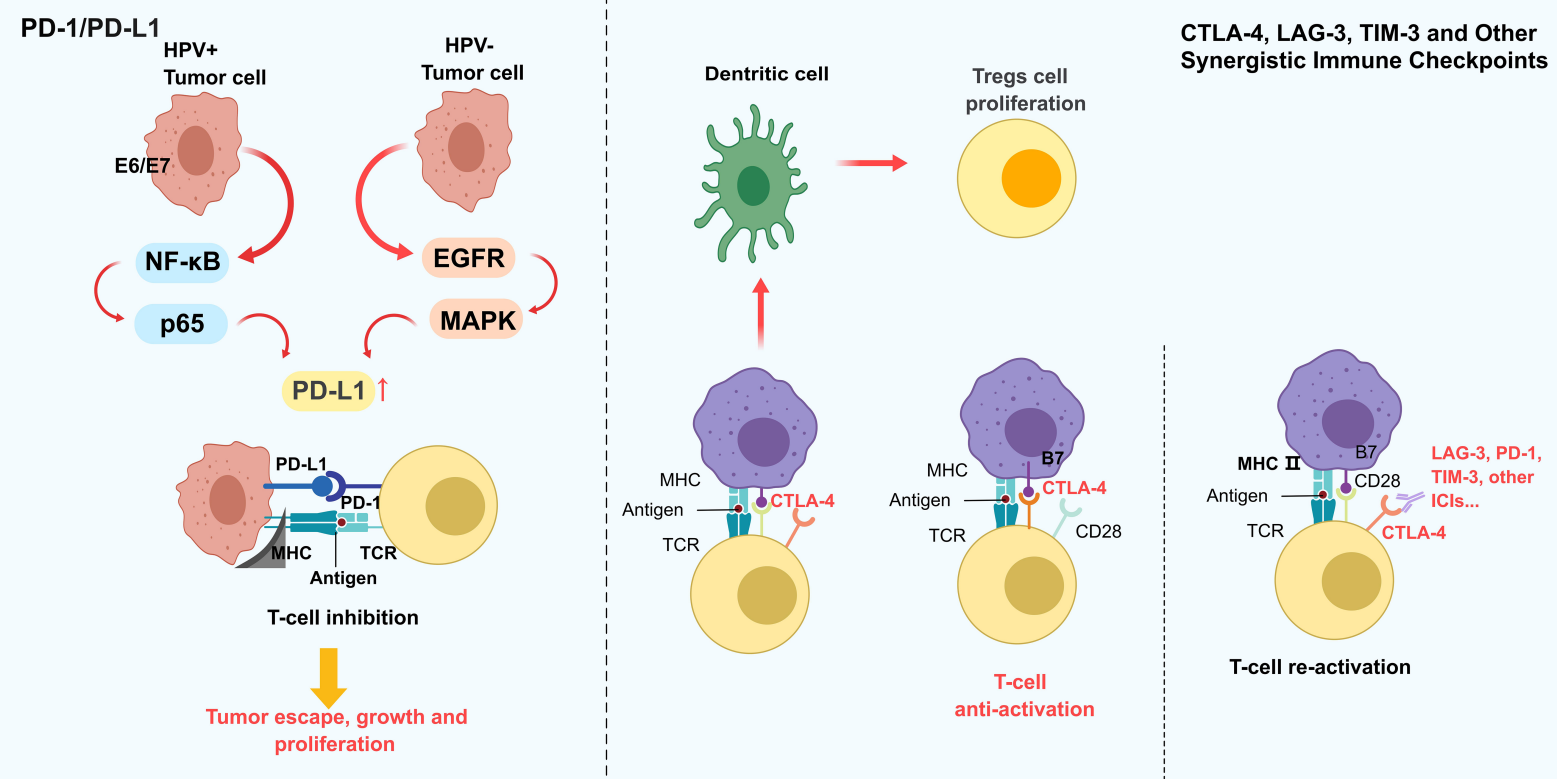
Dynamic expression and interaction patterns of different HNSCC immune checkpoints. The PD-1/PD-L1 axis as a central immune evasion mechanism in HNSCC, regulated by HPV status: HPV+ tumors upregulate PD-L1 via NF-κB/p65 signaling, while HPV− tumors rely on EGFR/MAPK pathways. PD-L1 binds to PD-1 on T cells, inducing T cell inhibition and promoting tumor escape. Other checkpoints, including CTLA-4, LAG-3, and TIM-3, further suppress T cell function by blocking co-stimulatory signals (e.g., CD28-B7) and enhancing Treg proliferation. This synergistic suppression creates a pro-tumorigenic microenvironment. The figure emphasizes the need for combination therapies targeting multiple checkpoints to overcome limitations of PD-1/PD-L1 monotherapy, which often face modest responses due to spatial heterogeneity and T cell exhaustion.

The PD-1/PD-L1 signaling axis, a central hub of immune evasion in HNSCC, exhibits marked spatiotemporal heterogeneity in its expression dynamics (**Figure 2**). Single-cell spatial analyses reveal that PD-L1 is specifically overexpressed in CD44+EpCAM+ cancer stem-like subpopulations at invasive tumor fronts, where it induces T cell inactivation through dual mechanisms: membrane-bound signaling and exosome-mediated delivery (36). This expression pattern correlates with HPV status (37), HPV+ tumors upregulate PD-L1 transcription via NF-κB/p65 signaling driven by E6/E7 oncoproteins, whereas HPV− tumors primarily rely on EGFR/MAPK pathways (15). PD-L1 is expressed in approximately 50% of HNSCC tumors, with higher prevalence in HPV+ cases (18). IFN-γ-induced PD-L1 regulation exhibits biphasic dynamics: acute stimulation rapidly upregulates expression via JAK/STAT signaling, while chronic exposure leads to persistent hypermethylation-associated expression (38). Despite the approval of PD-1/PD-L1 inhibitors for recurrent/metastatic HNSCC, clinical response rates remain modest (15–20%), attributed to spatial heterogeneity, the absence of tertiary lymphoid structures, CXCL13+CD8+ T cell exhaustion, and tumor mutational burden (39). These findings underscore the limitations of monotherapy and emphasize the need for combinatorial strategies targeting complementary immunosuppressive pathways.

### 3.2 Multidimensional regulation of synergistic immune checkpoints

CTLA-4, a critical early immune checkpoint, sculpts immunosuppressive niches via two mechanisms: (1) competitive B7 ligand binding in lymph nodes to inhibit naïve T cell priming, and (2) IDO+ dendritic cell-mediated Treg expansion within tumors (40). Preclinical evidence suggests that dual CTLA-4/PD-1 blockade enhances antitumor immunity. However, TIM-3 overexpression exacerbates PD-1-driven T cell exhaustion by suppressing IL-2 secretion in CD4+ T cells, while LAG-3 amplifies CTLA-4 suppression via MHC class II-dependent pathways (41). TIM-3 and LAG-3 further promote immune escape by impairing T cell function through distinct mechanisms (42). Despite these advances, clinical efficacy remains constrained by the redundancy of immune evasion mechanisms, necessitating rational combinations of checkpoint inhibitors and microenvironmental modulators.

### 3.3 Precision targeting strategies for immune checkpoints

Immune checkpoint inhibitors (ICIs), including anti-PD-1 antibodies such as pembrolizumab and nivolumab, are approved therapies for HNSCC, but anti-CTLA-4 antibodies are still under investigation and not yet considered standard of care (40, 43–47). Additionally, the metabolic reprogramming observed in HNSCC, including upregulation of glycolytic pathways and lactate production, contributes to an acidic microenvironment that suppresses T cell function and reduces the efficacy of ICIs (48). Macrophage reprogramming, such as depleting M2-polarized TAMs or blocking CSF-1/IL-10 signaling, reverses immunosuppression and synergizes with PD-1 blockade (49). Furthermore, epigenetic changes in tumor cells and immune cells can lead to a loss of PD-L1 expression or promote immune exhaustion, rendering T cells unresponsive to checkpoint blocked. Emerging therapeutic strategies targeting these resistance mechanisms aim to restore T cell function, such as by reprogramming TAMs or targeting key metabolic enzymes like lactate dehydrogenase (LDH), which has shown promise in enhancing the antitumor efficacy of ICIs. Moreover, combination therapies that incorporate epigenetic modulators to reverse immune evasion mechanisms are being actively explored as a way to overcome resistance to current therapies. These combined approaches hold potential for improving the overall success rates of immunotherapies in HNSCC and other solid tumors.

The PD-1/PD-L1 axis and synergistic checkpoints play pivotal roles in HNSCC immune evasion. Understanding their spatiotemporal regulation, compensatory networks, and microenvironmental interactions is key to developing next-generation immunotherapies. Rational combinations of checkpoint inhibitors with metabolic modulators, stromal disruptors, or epigenetic therapies hold promise for transforming immune “cold” HNSCC into responsive ecosystems, ultimately improving clinical outcomes.

## 4 Metabolic reprogramming and its impact on immune evasion

### 4.1 Hallmarks of tumor metabolic reprogramming

HNSCC exhibits spatially heterogeneous metabolic reprogramming that supports tumor survival and immune evasion. The classical “Warburg effect” (aerobic glycolysis) not only fuels ATP production but also generates an acidic microenvironment via lactate accumulation, especially in hypoxia in multiple cancers (50–52), directly suppressing CD8+ T cell cytotoxicity (53). Single-cell metabolomics reveals compartmentalized metabolic in HNSCC: glucose-dependent metabolism dominates at invasive margins, while core regions favor glutamine metabolism, creating dynamic gradients of metabolites (54). Lipid metabolism also facilitates immune escape, tumor cells utilize FABP4-mediated lipid droplet storage for energy reserves and secrete lipocalin-2 (LCN2) to polarize macrophages toward an M2 immunosuppressive phenotype (55). These reprogrammed pathways reshape the TME, depleting nutrients and altering metabolite profiles to suppress immune cell activity (10).

### 4.2 Immunosuppressive roles of metabolic by-products

Tumor-derived metabolites reinforce immune evasion through signaling and epigenetic remodeling. Lactate acts beyond metabolism—via GPR81 signaling, it induces dendritic cell tolerization and inhibits CD8^+^ T cell effector function by hyperacetylating the IFN-γ promoter through HDAC inhibition (38). Acidosis (pH 6.5–6.8) further impairs T cell proliferation and cytotoxicity (15). Additionally, lipid-derived mediators such as prostaglandin E2 (PGE2) promote M2 macrophage polarization and Treg expansion, consolidating the immunosuppressive niche (56).

### 4.3 Metabolic targeting strategies

Targeting tumor metabolism has emerged as a promising approach to enhance immunotherapy. Inhibiting glycolysis or lipid metabolism can restore T-cell function; for instance, targeting HK2 can reverse impaired glucose uptake in PD-1^+^ CD8^+^ T cells (53). Combination strategies are also under investigation: the adenosine receptor antagonist AB928, when paired with anti-PD-1 therapy, significantly improved responses in HPV-negative HNSCC by promoting TLS formation and increasing CXCR3^+^ T cell infiltration (57). Thus, metabolic interventions may overcome immune resistance and broaden treatment options. Continued research into how metabolic rewiring facilitates immune escape could yield novel therapeutic targets and optimize existing immunotherapies (58).

## 5 Current advances in immunotherapy

### 5.1 Clinical use of PD-1/PD-L1 inhibitors

PD-1/PD-L1 inhibitors have made significant progress in the treatment of HNSCC. In recent years, anti-PD-1 monoclonal antibodies such as pembrolizumab and nivolumab have been approved by the FDA and EMA for the treatment of patients with recurrent or metastatic HNSCC. By blocking the interaction between PD-1 and its ligand PD-L1, these drugs restore the anti-tumor activity of T cells, thereby enhancing the immune system ‘s attack on tumor cells. However, their overall clinical efficacy remains limited—approximately 60% of patients do not respond, and only 20–30% achieve durable progression-free survival (47).

Emerging evidence suggests that this limited efficacy is largely attributable to TME factors, including immune exclusion, upregulation of immunosuppressive cytokines, and downregulation of human leukocyte antigen (HLA) expression (40). For example, increased infiltration of MDSCs and regulatory T cells has been associated with resistance to anti-PD-1 therapy. To address these challenges, combination therapies are being actively explored. Clinical trials have demonstrated that combining PD-1/PD-L1 inhibitors with chemotherapy or radiotherapy can synergistically enhance antigen release and immune priming. In KEYNOTE-048, for instance, pembrolizumab plus chemotherapy significantly improved overall survival compared to chemotherapy alone in PD-L1–positive HNSCC. Preclinical models also support combinations with anti-angiogenic agents, epigenetic modulators (e.g., DNMT or HDAC inhibitors), or metabolic modulators targeting lactate dehydrogenase (LDH) and adenosine pathways.

Future directions include rational selection of patients based on biomarkers (TMB, PD-L1 expression, and TLS formation) and spatiotemporal profiling of immune infiltration to guide individualized therapy.

### 5.2 Current status and challenges of CAR-T cell therapy

CAR-T cell therapy is an emerging immunotherapeutic approach that has achieved significant success in hematological tumors, but its use in solid tumors such as HNSCC remains challenging. CAR-T cells are genetically engineered to express specific antigen receptors, thereby enhancing the recognition and killing ability of tumor cells. However, the tumor microenvironment of HNSCC is often highly immunosuppressive, which can limit the effectiveness of CAR-T cell therapy (59–61).

In HNSCC, CAFs secrete IL-6, TGF-β, and ECM-modifying proteins such as LOXL2, which restrict T cell trafficking and promote Treg polarization. Additionally, tumor antigen heterogeneity and antigen loss variants further compromise CAR-T targeting. CAFs showed inhibitory effects on CD8 + T cells, further limiting the antitumor activity of CAR-T cells (Qin, 9).

To overcome these obstacles, researchers are exploring multiple strategies, including optimizing the manufacturing process of CAR-T cells, combining other immunotherapies or targeted therapies, and developing novel CAR-T cell designs to improve their efficacy in solid tumors (62). These improvements are expected to overcome the limitations of existing treatments and enhance the clinical application of CAR-T therapy in HNSCC.

### 5.3 Exploration of other immunotherapy strategies

Beyond PD-1/PD-L1 inhibitors and CAR-T cell therapy, several alternative and adjunctive immune-therapeutic strategies are under rapid development in HNSCC. For example, interventions directed at the tumor microenvironment, such as by modulating the function of TAMs or CAFs, may effectively enhance antitumor immune responses (63, 64). In addition, cancer vaccines and immunomodulators are being developed to improve the immune response to tumors by activating the patient ‘s immune system (65). Combination regimens involving radiotherapy have shown synergistic effects, where ionizing radiation increases tumor antigen release and upregulates immune checkpoint molecules, thereby sensitizing tumors to PD-1 blockade (Chen, 66).

However, these strategies face several challenges: Lack of validated biomarkers to guide patient selection; Heterogeneity in immune phenotypes among HPV+ vs. HPV− tumors; Incomplete understanding of immune escape mechanisms in immune-desert tumors; Need for robust clinical data to confirm long-term efficacy and safety (67).

## 6 Future directions

### 6.1 Rational combination therapies

In the treatment of HNSCC, monotherapies often yield suboptimal outcomes, underscoring the need for rational combination strategies. Studies demonstrate that multimodal approaches can overcome immune evasion mechanisms in the TME, thereby enhancing therapeutic efficacy (47). Combining immune checkpoint inhibitors with chemotherapy, radiotherapy, or targeted therapies can amplify antitumor immune responses. Additionally, cancer stem cells significantly contribute to metastasis and immune evasion, making CSC-targeting approaches a critical component of combination regimens (19).

Specific combinations, such as PD-1/PD-L1 inhibitors co-administered with other immunomodulators (TGF-β blockers or CSF-1R inhibitors), show promise in reshaping the immunosuppressive TME. For instance, dual PD-1/CTLA-4 blockade has yielded improved response rates and survival outcomes in HNSCC patients (15).

Future clinical trials could explore that sequential or adaptive regimens that tailor dosing intervals and drug sequencing to dynamic TME changes, and combinations with epigenetic therapies or metabolic modulators to restore tumor immunogenicity and T cell function. This comprehensive approach may bolster the durability of immunotherapy responses and overcome primary or acquired resistance (68, 69). These strategies aim to comprehensively reshape the immunosuppressive TME, offering new avenues for improving immunotherapy outcomes.

### 6.2 Development of personalized treatment strategies

Personalized therapy has become essential for optimizing HNSCC treatment outcomes. With the advent of omics technologies, clinicians can now profile tumor genetics, proteomics, and immunophenotypes to tailor individualized regimens. For instance, TMB and TME characteristics have been identified as predictive biomarkers for immunotherapy response (70). Integrating genomic profiling of tumor samples allows for precision immunotherapy selection, targeting specific mutations and expression patterns (71).

In addition to genetic and molecular data, clinical features such as HPV status and comorbidities also inform personalized treatment. PD-L1 expression levels can guide therapeutic decisions regarding PD-1/PD-L1 inhibitors (72) (18). Moving forward, prospective clinical trials should incorporate longitudinal biomarker analysis—such as monitoring circulating tumor DNA (ctDNA) or single-cell transcriptomic changes—to refine therapy choices in real time.

### 6.3 Discovery and application of biomarkers

Biomarker development is central to early diagnosis, prognostication, and treatment monitoring in HNSCC. Recent high-throughput methods, including single-cell RNA sequencing and bioinformatics-driven integrative analyses, have revealed numerous biomarkers correlated with tumor progression and immune evasion (73). For instance, dysregulated long non-coding RNAs (lncRNAs) may predict immunotherapy responses and disease trajectory (74).

Future research should focus on multiparametric biomarker panels, integrating immune cell composition, cytokine profiles, and metabolic signatures to stratify patients more accurately. In addition to that, TAM and CAF phenotypes, as their polarization states strongly impact patient prognosis and therapy responsiveness (75, 76). By combining multi-biomarker analyses with functional TME characterization, we can drive the evolution of precision medicine. Large-scale clinical trials, employing adaptive designs and real-time biomarker assessment, will accelerate the translation of these findings into standard-of-care practices.

## 7 Conclusion

The immune escape mechanisms in HNSCC represent a pivotal focus in tumor biology and immunotherapy research. As our understanding of the TME expands, it has become clear that tumor cells do not exist in isolation but engage in dynamic interactions with immune cells, stromal components, and the extracellular matrix. These interactions play a profound role in influencing tumor growth, metastasis, and therapeutic responses.

This review emphasizes the critical role of immune cell infiltration patterns and checkpoint dysregulation in the progression of HNSCC. While high infiltration of CD8+ T cells is generally correlated with favorable clinical outcomes, TAMs and Tregs are frequently associated with tumor progression and poor prognosis. However, inconsistencies in study outcomes, influenced by variations in sample selection, methodologies, and analytical approaches, pose significant challenges to integrating findings. Addressing these discrepancies is crucial for advancing HNSCC immunotherapy.

Recent advances in immunotherapy, particularly immune checkpoint inhibitors, have demonstrated considerable clinical benefits. By restoring immune activity through PD-1/PD-L1 or CTLA-4 blockade, these therapies have shown promise in improving patient survival and quality of life. Nevertheless, their efficacy remains variable, highlighting the urgent need for strategies to identify the appropriate patient populations and develop personalized treatment regimens.

Looking ahead, the complexity of the TME offers numerous opportunities for future research. Investigating the intricate interactions between tumor cells and immune cells, and exploring methods to modulate the TME, such as targeting immunosuppressive cytokines or employing genetic editing to alter tumor cell immunophenotypes, holds great potential for enhancing the efficacy of immunotherapy. Additionally, a deeper understanding of these mechanisms could inform the development of combination therapies, further improving patient outcomes.

In conclusion, the immune escape mechanisms in HNSCC reflect the intricate interplay between tumors and their microenvironment. Future research should focus on clarifying these complex interactions and identifying biomarkers for personalized treatment strategies. Multidisciplinary collaboration, integrating basic research with clinical trials, will be essential in driving the development of more effective therapies. These advances not only offer hope for improved HNSCC outcomes but also provide valuable insights for immunotherapy across a range of other malignancies, setting the stage for broader clinical applications.
